# Immunotherapy: rAAV2 expressing interleukin-15 inhibits HeLa cell tumor growth in mice

**DOI:** 10.1186/1423-0127-16-47

**Published:** 2009-05-07

**Authors:** Giou-Teng Yiang, Horng-Jyh Harn, Yung-Luen Yu, Sheng-Chuan Hu, Yu-Ting Hung, Chia-Jung Hsieh, Shinn-Zong Lin, Chyou-Wei Wei

**Affiliations:** 1 Institute of Medical Sciences, Tzu Chi University, Hualien, Taiwan; 2 Department of Emergency Medicine, Tzu Chi General Hospital, Hualien, Taiwan; 3 Department of Pathology, China Medical University Hospital, Taichung, Taiwan; 4 Graduate Institute of Cancer Biology and Center for Molecular Medicine, China Medical University and Hospital, Taichung, Taiwan; 5 Department of Biotechnology, Asia University, Taichung, Taiwan; 6 Institute of Biomedical Nutrition, College of Medicine & Nursing, Hung Kuang University, Sha Lu, Taichung, Taiwan; 7 Department of Neurosurgery, China Medical University Hospital, Taichung, Taiwan

## Abstract

Human interleukin-15 (hIL15) has anti-tumor activities, but it is not convenient for tumor treatment because of its short half-life. A gene therapy for mouse lung cancer using an adenovirus vector expressing IL15 has been reported. However, adenovirus vector-mediated gene therapy can provoke cellular toxicity and inflammatory reactions. The recombinant adenovirus-associated vector 2 (rAAV2) is safer due to minimal cellular toxicity and immune response. In order to demonstrate that gene therapy can be used safely and successfully for human cancer treatment, the rAAV2 expressing hIL15 gene (rAAV2-hIL15) is applied for human cervical cancer, HeLa cell, in this study. This study successfully demonstrates that rAAV2-hIL15 can express IL15 with bioactivities in vitro and in vivo. In conclusion, our studies show that human cervical cancers are inhibited on animal model with rAAV2-hIL15 treatment and provide a safer and important reference for human cancer gene therapy.

## Background

There had been many studies that considered immunotherapy as a potent method for the treatment of cancer and this method inhibits tumor growth [1,2]. Both interleukin-2 (IL2) and interleukin-15 (IL15) have been used for cancer immunotherapy [3,4]. However, previous studies indicated that IL2 displayed a significant toxicity in immunotherapy after systemic administration of high-dose IL2 [5,6]. IL15 is a 14–15 Kd immunostimulatory cytokine which belongs to a four α-helix cytokine family [7,8]. Using IL15 for cancer immunotherapy based on NK cell activity and immunoglobulin production had been demonstrated previously [9,10]. Repeated daily injection is required because of the short biological half-life using IL15 administration [11-13]. In addition, some studies showed that tumor cells engineered to secrete IL15 can inhibit tumor growth on animal models [6,14]. That would suggest that replacing daily administration using gene therapy may be useful for cancer immunotherapy. Recently, a study demonstrated that an adenovirus vector expressing IL15 gene can inhibit lung cancer in mouse [15]. However, adenovirus vector is not suitable for human gene therapy due to its cytoxicity and inflammatory response [16]. Hence this study used a safer viral vector (rAAV2) expressing hIL15 for human cervical cancer treatment.

Many studies have demonstrated that rAAV2 is a good choice for gene therapy because of the attractive features offered by this gene delivery system. The major advantages of rAAV2's are nonpathogenic in human with low immunogenicity compared with the other viral delivery systems [17,18]. Moreover, rAAV2s can infect both the dividing and quiescent cells by inducing a long-term stable gene expression in a wide variety of tissues [19-21]. Recently, rAAV2 has been demonstrated safe in human clinical trails [22,23]. Based on these attractive features, we chose rAAV2 delivery system to express hIL15 for cancer immunotherapy.

Previous study has indicated that rAAV2 vector is difficult to scale up [24]. In this study, we used the helper-free system to produce rAAV2 expressing human IL15 gene (rAAV2-hIL15) [25] and purified the rAAV2-hIL15 with a single-step gravity-flow column (SSCP method) [26]. The purified rAAV2-hIL15 could exert bioactivities in vitro and in vivo. Furthermore, our data demonstrated that single intramuscular injection of rAAV2-hIL15 can effectively inhibit HeLa cells growth on nude mouse model. This is the first research study that used a safe viral vector rAAV2 expressing human IL15 gene for cancer immunotherapy and successfully inhibited tumor cell growth on an animal model. This study can provide a safe and effective reference for cancer gene therapy in the future

## Materials and methods

### Culture Conditions

HeLa (human cervical cancer), 293 (human embryonic kidney) and HT1080 (human fibrosarcoma) cells were purchased from Bioresource Collection and Research Center (BCRC, Taiwan) and cultured in Dulbecco's modified Eagle's medium (DMEM) supplemented with 10% fetal bovine serum, 2 mM L-glutamine, 100 IU/ml penicillin/streptomycin, 1 mM sodium pyruvate, and 0.1 mM non-essential amino acids. HT2 (murine IL2/IL15 dependent) cells were purchased from BCRC and cultured in RPMI 1640 medium supplemented with 10% fetal bovine serum, 2 mM L-glutamine, 100 IU/ml penicillin/streptomycin, 1 mM sodium pyruvate, and 0.1 mM non-essential amino acids. In addition, HT2 cells were supplemented with 1 μg/ml human recombinant IL15 protein (Santa Cruz Biotechnology, Santa Cruz, California, USA). DMEM, RPMI, fetal bovine serum, L-glutamine, penicillin/streptomycin, sodium pyruvate and non-essential amino acids were bought from Invitrogen (Carlsbad, California, USA).

### Plasmids and Plasmid Construction

The rAAV2 helper-free system, containing packaging plasmid pAAV-MCS plasmid, pAAV-RC plasmid, pHelper, pAAV-hrGFP plasmid was purchased from Strategene (La Jolla, California, USA). The plasmid containing human IL15 gene (hIL15) was provided thru the kindness of Dr L. KW (National Chiao Tung University, Taiwan). The hIL15 containing EcoR I and BamH I sites was amplified for cloning using the polymerase chain reaction. Oligonucleotide primers containing EcoR I and BamH I sites were synthesized as follows: sense primer: 5'GAATTC AAA GAA TTC ATG TAC AGG ATG CAA CTC CT, and anti-sense primer, 3'GGATCC AAA GGA TCC TTA AGA AGT GTT GAT GAA CAT TTG G. The PCR conditions used a thermal profile of denaturing at 95°C for 30 sec, annealing at 95°C for 30 sec, and extending at 72°C for 30 sec for 30 cycles, followed by 72°C for 10 min. The amplified hIL15 cDNA was inserted between the EcoR I and BamH I sites of pAAV-MCS to yield pAAV-hIL15 plasmid.

### Production of rAAV2

Production of rAAV2-hIL15 and rAAV2-hrGFP was done in the helper-free system as described previously [25]. For rAAV2-hIL15 production, 293 cells were cultured on fifty 15-cm-dishes and transfected with calcium chloride with 2 mg pAAV-hIL15 plasmid, 2 mg pAAV-RC plasmid and 2 mg pAAV-Helper plasmid. For rAAV2-hrGFP production, 293 cells were cultured on fifty 15-cm-dishes and transfected with calcium chloride with 2 mg pAAV-hrGFP plasmid, 2 mg pAAV-RC plasmid and 2 mg pAAV-Helper plasmid. After 65 hours of transfection, rAAV2-hIL15 and rAAV2-hrGFP were produced in 293 cells.

### Purification of rAAV2

Purification of rAAV2-hIL15 and rAAV2-hrGFP was done using a single-step column purification (SSCP) method [26]. The rAAV2 was concentrated to about 1 ml with an Amicon Ultra-15 centrifugal filter (Millipore, Billerica, MA) and stored at -80°C. Finally, the viral titers of rAAV2 were determined by Real-time PCR method as described previously [24]. A total of 2–5 × 10^12 ^genomic particles of rAAV2 can be obtained from fifty 15-cm-dishes. Purified rAAV2 (rAAV2-hIL15 and rAAV2-hrGFP) was determined by loading 10^10 ^viral particles on 10% SDS gel [26] and viral proteins (VP1, VP2 and VP3) were detected by silver stain

### Quantification of IL15 Expression

HT1080 cells can be infected with AAV2 easily for the production of AAV2 expressing genes. In this study, HT1080 (1.5 × 10^5^) cells were divided into three groups and cultured on 6-well-plates, then each group was treated with 100 μl of rAAV2-hIL15 (10^13 ^viral particles/ml), rAAV2-hrGFP (10^13 ^viral particles/ml) and PBS, respectively. After 2 days, the media were collected for quantification of IL15. IL15 titers were determined using an ELISA kit (Biosource, Camarillo, California, USA).

### In Vitro Bioactivity Assay

HT2 cells are IL2/IL15 dependent cells and can not survive without IL2 or IL15 supply. Therefore growth of HT2 cells is determined by the IL15 bioactivity. HT2 cells were divided into three groups and their survival rates were determined by using MTS assay (Promega, Madison, Wisconsin, USA) under an optical density 490 nm. The first group of HT2 cells was cultured with the media obtained from 100 μl of rAAV2-hIL15 (10^13 ^viral particles/ml)-infected HT1080 cells. The second group was cultured with the media obtained from 100 μl of rAAV2-hrGFP (10^13 ^viral particles/ml)-infected HT1080 cells. The third group of HT2 cells was cultured with the media obtained from purified 1 μg/ml human recombinant IL15-incubated HT1080 cells (This group was used as the positive control). These were then observed under the microscope.

### In Vivo Bioactivity Assay of rAAV2-hIL15

Nude mice are T-cell defective in which NK cell activity cannot be determined. Previous research showed that the increase in the immunoglobulin titer can be an indication of an increase in the IL15 activity [10]. In addition, it has been reported that this immunoglobulin is related to antitumor activity on nude mice [27]. Therefore immunoglobulin level was determined in this study. Twenty four (24)-four-week-old female nude mice were purchased from National Laboratory Animal Center (Taiwan). All mice were divided into three groups (8 mice/each group). The first group of mice was injected with 500 μl of rAAV2-hIL15 (10^13 ^viral particles/ml) on one site of the quadriceps muscles of their hind limbs, the second group with 500 μl of rAAV2-hrGFP (10^13 ^viral particles/ml) and the third group with 500 μl of PBS (the steps and the amount injected were the same as the first group). After 28 days, the mice sera were collected for immunoglobulin measurement. Quantification of immunoglobulin levels was performed using immunoglobulin ELISA kit (Becton, Dickinson and Company) measured at an optical density 450 nm. The animal studies have been approved by the animal committee of the Tzu-Chi General Hospital.

### Determination of Mice Weight

Twenty four, four-week-old female nude mice were purchased from National Laboratory Animal Center (Taiwan). All mice were divided into three groups (8 mice/group). The first group of mice was injected with 500 μl of rAAV2-hIL15 (10^13 ^viral particles/ml) on one site of their quadriceps muscles of the hind limbs, the second group with 500 μl of rAAV2-hrGFP (10^13 ^viral particles/ml) and the third group with 500 μl of PBS (the steps and amount injected were the same as the first group). Every week, the weight of the treated-mice was determined using electronic weighing-scale. The animal studies have been approved by the animal committee of the Tzu-Chi General Hospital.

### Animal Study I (Pre-treated with rAAV-IL15)

Twenty four, four-week-old female nude mice were purchased from National Laboratory Animal Center (Taiwan). All mice were divided into three groups (8 mice/group). The first group of mice was injected with 500 μl of rAAV2-hIL15 (10^13 ^viral particles/ml) on one site of quadriceps muscles of their hind limbs, the second group with 500 μl of rAAV2-hrGFP (10^13 ^viral particles/ml) and the third group with 500 μl of PBS (steps and amount injected were the same as the first group). After 28 days, 10^7 ^HeLa cells were injected subcutaneously on the lower abdominal region of all mice. The day when mice began treatment with HeLa cells corresponded to day zero. After that, tumor size was measured every week according to the following formula: (A × B^2^)/2, where A is the greater and B is the smaller measurement of the two dimensions [28,29]. The animal studies have been approved by the animal committee of the Tzu-Chi General Hospital.

### Animal Study II (Post-treated with rAAV-IL15)

Twenty four, four-week-old female nude mice were purchased from National Laboratory Animal Center (Taiwan). All mice were divided into three groups (8 mice/group) and were injected with 10^7 ^HeLa cells on the lower abdominal region. The next day, the first group of mice was injected with 500 μl of rAAV2-hIL15 (10^13 ^viral particles/ml) on one site of quadriceps muscles of the hind limbs, the second group with 500 μl of rAAV2-hrGFP (10^13 ^viral particles/ml) and the third group with 500 μl of PBS (steps and amount injected were the same as the first group). The day when mice began treatment with HeLa cells corresponded to day zero. The, tumor size was then measured according to the following formula: (A × B^2^)/2, where A is the greater and B is the smaller measurement of the two dimensions [28,29]. The animal studies have been approved by the animal committee of the Tzu-Chi General Hospital.

### Statistical Analysis

The Student t-test was utilized in the analysis of data presented as the mean ± SE. A value of *P *< 0.05 was considered statistically significant.

## Results

### rAAV2-hIL15 Can Express IL15 Having Bioactivity in vitro

**Figure 1:**
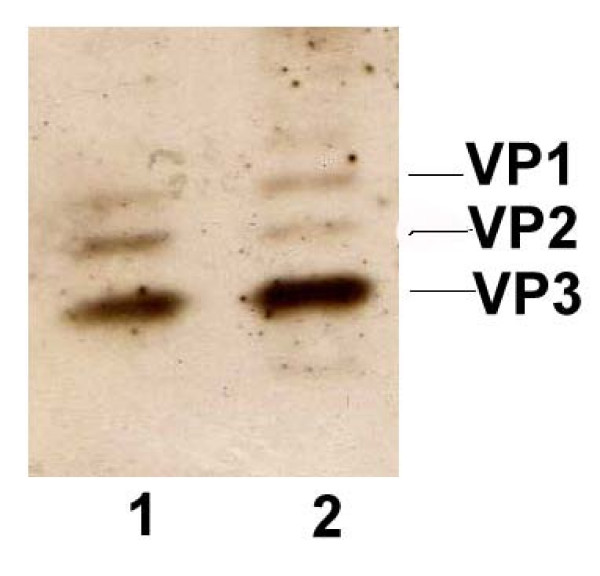
**Purification of hIL15**. 10^10 ^viral particles were determined on 10% SDS page with silver staining. Lane 1 showed rAAV2-hIL15 and lane 2 showed rAAV2-hrGFP.

**Figure 2:**
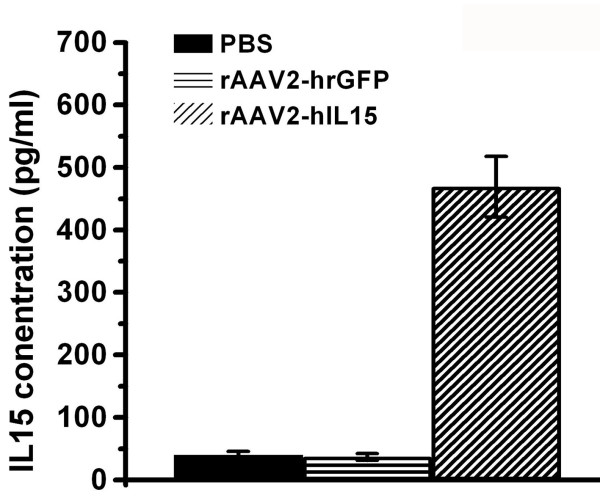
**Production of hIL15**. H1080 cells were treated with rAAV2-hIL15, rAAV2-hrGFP or PBS. After 2 days, media were collected and assayed for hIL15 using an ELISA kit. Data were obtained from three independent triplicate experiments and were presented as mean ± S.D.

**Figure 3:**
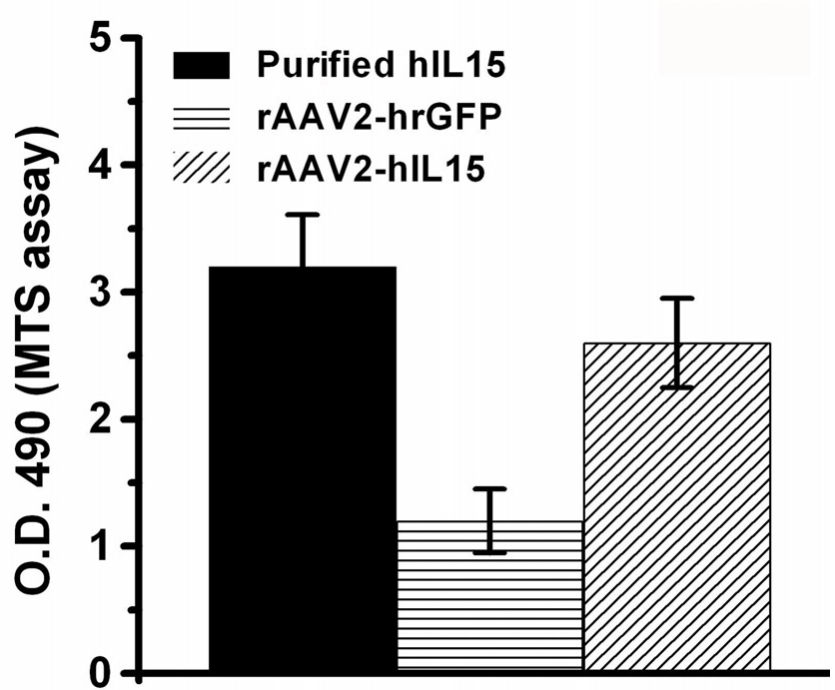
**Survival rate of HT2 cells**. Media obtained from rAAV2-hIL15-infected or rAAV2-hrGFP-infected HT1080 cells were added to HT2 cells culture. Media containing 1 μg/ml purified hIL15 were added to HT2 cells as a positive control. After 3 days, survival rate of HT2 cells was determined using MTS assay. Data were obtained from three independent triplicate experiments and were presented as mean ± S.D.

**Figure 4:**
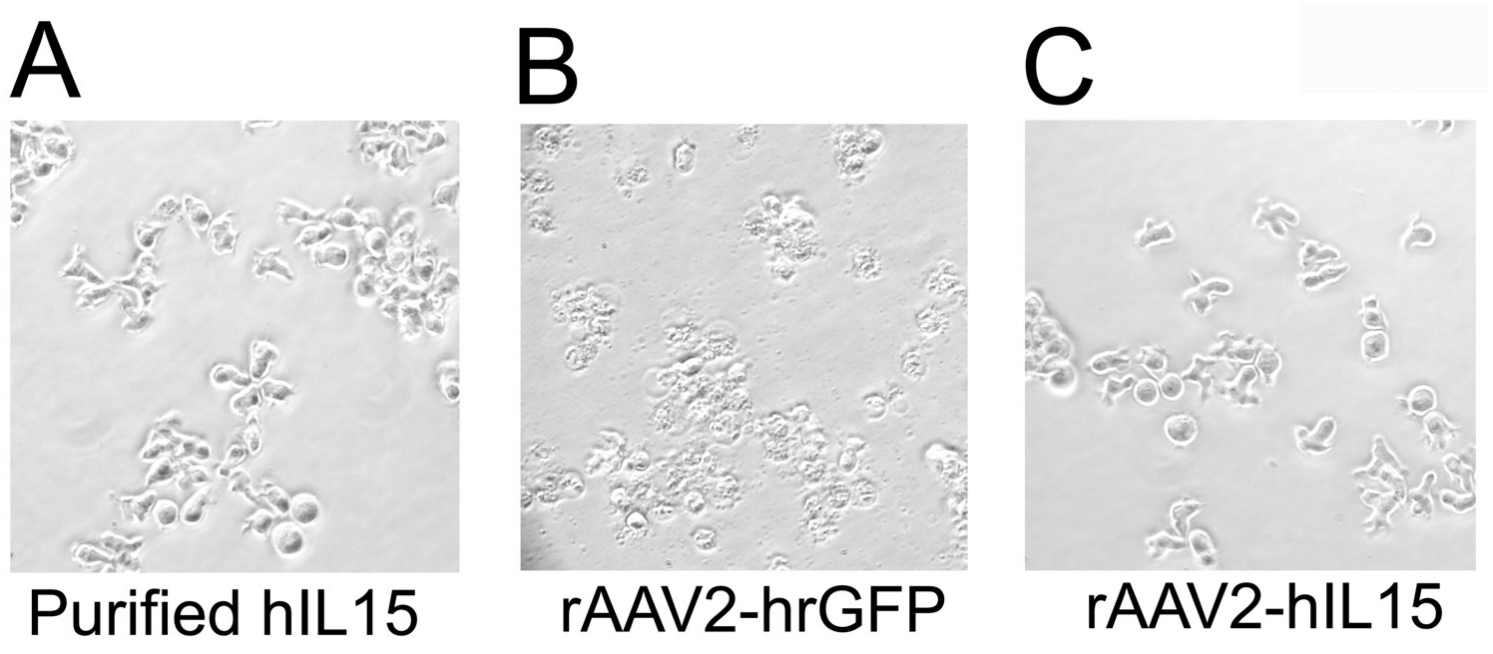
**Morphology of HT2 cells under a microscope**. HT2 cells were cultured for 3 days with media obtained from: 1 μg/ml purified hIL15 (A), rAAV2-hrGFP infected-HT 1080 cells (B), and rAAV2-hIL15 infected-HT 1080 cells (C).

With the use of a helper-free system and SSCP method, rAAV2-hIL15 and rAAV2-hrGFP were produced and purified successfully. As shown in Figure 1, purified rAAV2-hIL15 and rAAV2-hrGFP have VP1, VP2 and VP3 of AAV2 capsid proteins. This result is similar to previous study [26]. HT1080 cells can be infected with AAV2 easily for production of AAV2 expressing genes. Purified rAAV2-hIL15, rAAV2-hrGFP and PBS were added to HT1080 cells, respectively. After 2 days, the culture media of HT1080 cells were collected. For quantification of IL15, our data showed that the media obtained from rAAV2-hIL15 treated-HT1080 cells have IL15 of about 500 ng/ml (Figure 2). However the media obtained from rAAV2-hrGFP or PBS treated-HT1080 cells have less and less IL15 than rAAV2-hIL15 treated-cells (Figure 2). The data indicated that rAAV2-hIL15 can express IL15. HT2 cells are IL2/IL15 dependent cells and they cannot survive without IL15 (or IL2) supply. Therefore the survival rate of HT2 cells can be used as an indicator for IL15 bioactivities. The media obtained from rAAV2-hIL15, rAAV2-hrGFP-treated HT1080 cells were collected and added to HT2 cells respectively. The purified human IL15 added to HT2 cells was used as a positive control. Observation on HT2 survival rate, the data showed that HT2 cells can grow with the media containing purified human IL15 and with the media obtained from rAAV2-hIL15-treated HT1080 cells (Figure 3). However, media obtained from rAAV2-hrGFP-treated HT1080 cells resulted in survival inhibition on HT2 cells (Figure 3). The morphologies of HT2 cells were also observed under the microscope (Figure 4). The morphologic data indicated that HT2 cells were able to survive under the media containing purified human IL15 and the media obtained from rAAV2-hIL15-treated HT1080 cells. However, HT2 cells were not able to survive under the media obtained from rAAV2-hrGFP-treated HT1080 cells (this result has been demonstrated in figure 3). Taken together, we successfully produced and purified rAAV2-hIL15 which has bioactivity in vitro.

### rAAV2-hIL15 Induces Immunoglobulin Production in Mice

**Figure 5:**
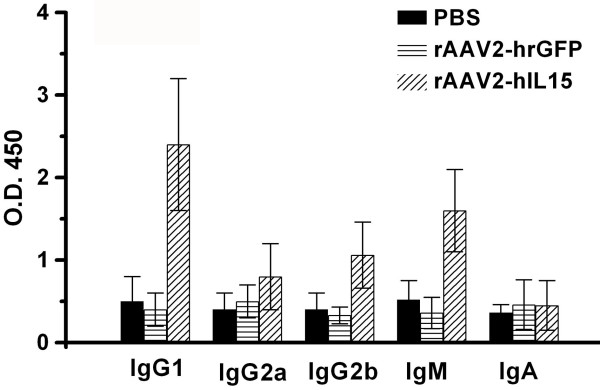
**Immunoglobulin levels**. Sera obtained from rAAV2-hIL15-treated, rAAV2-hrGFP-treated and PBS-treated mice for 28 days were examined, respectively. Quantification of isotype levels was performed using the ELISA method. Eight mice were determined for each group.

**Figure 6:**
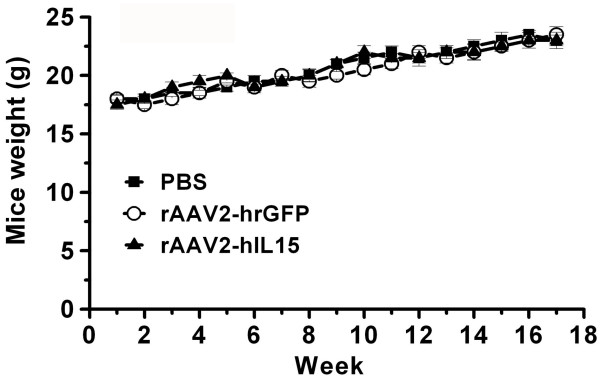
**Growth rate of animals**. All mice were treated with rAAV2-hIL15, rAAV2-hrGFP and PBS respectively and the mice weight was determined for 18 weeks. Each group used eight mice for weight determination.

Sera were collected from mice that were treated by intramuscular injection of rAAV2-hIL15, rAAV2-hrGFP and PBS for 28 days. The sera were analyzed for the production of immunoglobulins (IgG1, IgG2a, IgG2b, IgA, and IgM). The data showed that sera obtained from rAAV2-hIL15-treated mice had higher titers of IgG1 and IgM than sera obtained from rAAV2-hrGFP-treated and PBS-treated mice (Figure 5). However, production of IgG2a, IgG2b and IgA has no significant difference in rAAV2-hIL15-treated, rAAV2-hrGFP-treated and PBS-treated mice. The data suggested that rAAV2-hIL15 can induce production of IgG1 and IgM in nude mice. Furthermore, we examined whether rAAV2-hIL15 and rAAV2-hrGFP interfered with mice growth. Our data indicated that during the 18 weeks, there was no difference in growth rate among mice treated by rAAV2-hIL15, rAAV2-hrGFP, and PBS (Figure 6).

### Pre-treated rAAV2-hIL15 Inhibits Tumor Growth Efficiently

**Figure 7:**
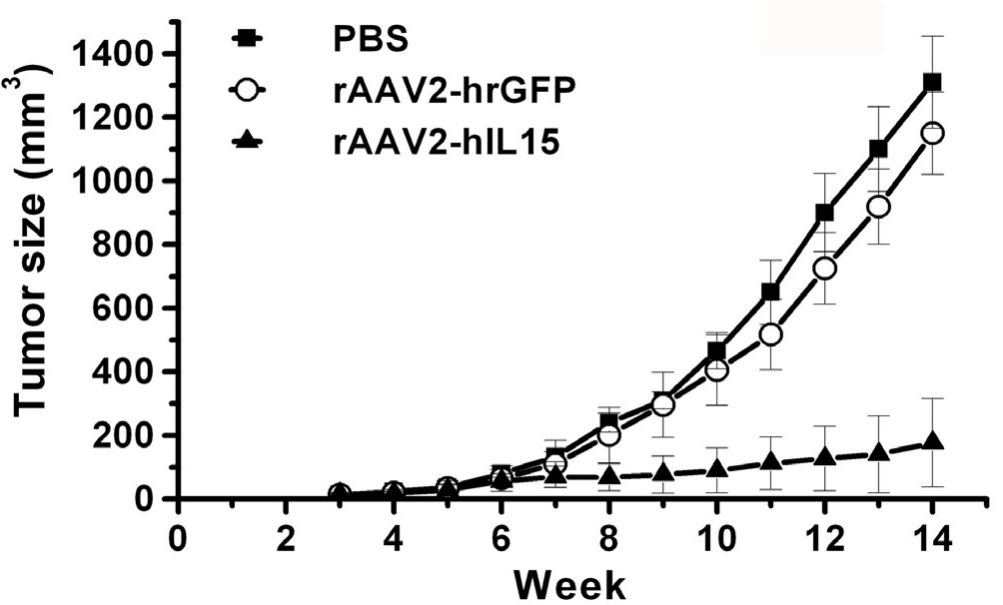
**Tumor volume of HeLa cervical cancers in pre-treated mice**. Mice were injected I.M. with rAAV2-hIL15, rAAV2-hrGFP and PBS respectively. After 28 days, HeLa cells were injected S.C. into these mice indicating day zero. Every week tumor size of these mice was determined. Eight mice were determined for each group.

Previous study had demonstrated that AAV, a single-stranded DNA virus, must convert single-stranded DNA into double stranded templates for transcription [30,31]. This rate-limiting step is achieved in about 3 weeks. Therefore, we first prescribed rAAV2-hIL15 before tumor implantation in the mice in this study. After rAAV2-hIL15, rAAV2-hrGFP and PBS were injected into the muscles of mice for 28 days, these mice received HeLa tumor cells implantation by subcutaneous injection. After which, tumor size was determined every week. Our data showed that tumor growth rate in rAAV2-hIL15-treated mice was slower than that in rAAV2-hrGFP- and PBS-treated mice (Figure 7). Meanwhile, tumor growth rates are similar in rAAV2-hrGFP-treated and PBS-treated mice. Comparing the tumor size on rAAV2-hIL15-treated mice with rAAV2-hrGFP- and PBS-treated mice, the difference in the size of the tumor becomes greater after the ninth week. These data indicated that pre-treated rAAV2-hIL15 could suppress tumor growth efficiently.

### Post-treated rAAV2-hIL15 Delays Tumor Growth

**Figure 8:**
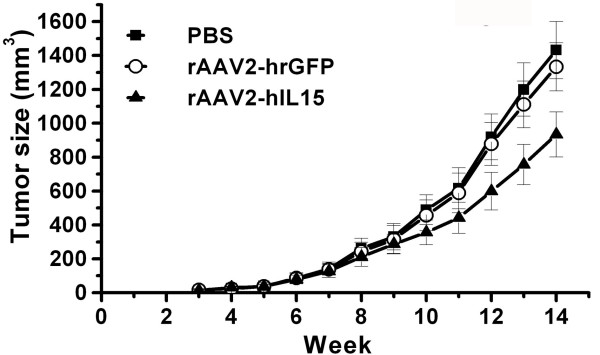
**Tumor volume of HeLa cervical cancers in post-treated mice**. HeLa cells were injected S.C. into the mice indicating day zero. Next day, rAAV2-hIL15, rAAV2-hrGFP and PBS were injected I.M. to the mice, respectively. Tumor size was determined weekly. Eight mice were determined for each group.

We examined next whether post-treated rAAV2-hIL15 has an anti-tumor effect. HeLa cells were first injected subcutaneously. in the lower abdominal region of the mice. The next day, these mice were given intramuscular injection of rAAV2-hIL15, rAAV2-hrGFP and PBS respectively. Before the 12th week, tumor sizes had no significant differences, but we found out that the tumor size was smaller in rAAV2-hIL15 treated-mice than in rAAV2-hrGFP-treated-mice and PBS-treated mice after 12 weeks (Figure 8). The result indicated that post-treated rAAV2-hIL15 could delay tumor growth. Altogether, our studies suggested pre-treated and post-treated rAAV2-hIL15 are a good approach for cancer gene therapy, yet pre-treated rAAV2-hIL15 is more efficient in inhibiting tumor growth.

## Discussion

Previous studies have shown that tumors were inhibited effectively by IL2 or IL15 due to NK cells activation and immunoglobulins secretion [9,10]. IL15 engineered tumor cells or daily administration of IL15 can suppress tumor growth has been demonstrated previously [6,11-14]. However, daily administration of IL15 is not convenient for cancer treatment because the life span of IL15 is short, and thus IL15 must be given by i.m. injection for many days [12,13]. In order to use IL15 for cancer treatment and to avoid treating IL15 many times, gene therapy is considered. Previous study has used adenoviral vector expressing IL15 gene for mouse lung cancer treatment [15]. Although adenovirus vector expressing IL15 gene can inhibit mouse lung cancer, it is difficult to be applied on human cancer treatment due to adenovirus vector-induced cytotoxicity and inflammatory response [16].

Many gene delivery systems have been developed [24,32-34]. rAAV2 is one of delivery systems for gene therapy and has many attractive features including long-term transgene expression, low immunogenicity, and lack of apparent pathogenicity [17,18]. Though rAAV2 is a small viral vector and can only carry about 5000 nucleotides gene sequence [35], human IL15 gene (about 600 nucleotides) can be packaged into rAAV2 vector for tumor treatment. In addition, previous studies showed that muscle can be used to produce therapeutic proteins encoded by infected with rAAV2 [36,37]. Northern blot has demonstrated that expression of IL15 mRNA can be observed in skeletal muscle [7,38]. Therefore, rAAV2-hIL15 was injected into the quadriceps muscle of mice in this study.

The study showed that pre-treated rAAV2-hIL15 can inhibit cervical cancer growth effectively (Figure 7). However, inhibition of tumor growth was less efficient by post-treated with rAAV2-hIL15 (Figure 8). Previous reports indicated that AAV is a single-strand DNA virus and must convert single-strand DNA into double strand template for transcription [30,31]. This rate-limiting step takes about 3 weeks. These previous results indicated that AAV-hIL15 can express IL15 after 21 days with AAV-IL15 treatment in our study. That is, IL15 has been expressed before tumor transplantation in our pre-treated rAAV2-hIL15 study, but IL15 was not expressed before 3 weeks with tumor transplantation in our post-treated rAAV2-hIL15 study. Therefore inhibition of tumor growth was more efficient when pre-treated with rAAV2-hIL15 for 28 days while tumor inhibition was less efficient when post-treated with rAAV2-hIL15. We considered that rAAV2 converted single-strand DNA into double strand template at the moment. To sum up, conversion of rAAV2 single-strand DNA into double strand template for gene expression is very important for gene therapy [31,39]. Based on this reason, we suggest that using a new rAAV2 vector containing a double-strand DNA genome may overcome this problem [39]. Additionally, to increase the anti-tumor effect of rAAV2-hIL15 treatment, combination with other therapies (such as chemotherapy, radiotherapy, surgical treatment) will be considered on clinical treatment in the future.

## Competing interests

The authors declare that they have no competing interests.

## Authors' contributions

GTY, HJH, SCH and WCW conducted animal studies, immunochemistry experiments and AAV production. YLY, YTH and CJH performed bioactivity assay, morphology observation and gene construction. HJH, SZL and WCW participated in design of the study. YLY and WCW wrote the manuscript. All authors read and approved the final manuscript.
